# Molecular, biochemical and kinetic analysis of a novel, thermostable lipase (LipSm) from *Stenotrophomonas maltophilia* Psi-1, the first member of a new bacterial lipase family (XVIII)

**DOI:** 10.1186/s40709-018-0074-6

**Published:** 2018-02-08

**Authors:** Maria Parapouli, Athanasios Foukis, Panagiota-Yiolanda Stergiou, Maria Koukouritaki, Panagiotis Magklaras, Olga A. Gkini, Emmanuel M. Papamichael, Amalia-Sofia Afendra, Efstathios Hatziloukas

**Affiliations:** 1 Enzyme Biotechnology and Genetic Engineering Group, University of Ioannina, 451 10 Ioannina, Greece; 2 Department of Biological Applications & Technologies, University of Ioannina, University Campus, 451 10 Ioannina, Greece

**Keywords:** Thermostable bacterial lipase, *Stenotrophomonas maltophilia*, Lipase gene cloning and overexpression, Enzyme purification, Enzyme kinetics, Bacterial lipase families

## Abstract

**Background:**

Microbial lipases catalyze a broad spectrum of reactions and are enzymes of considerable biotechnological interest. The focus of this study was the isolation of new lipase genes, intending to discover novel lipases whose products bear interesting biochemical and structural features and may have a potential to act as valuable biocatalysts in industrial applications.

**Results:**

A novel lipase gene (*lipSm*), from a new environmental *Stenotrophomonas maltophilia* strain, Psi-1, originating from a sludge sample from Psittaleia (Greece), was cloned and sequenced. *lipSm* was further overexpressed in *E. coli* BL21(DE3) and the overproduced enzyme LipSm was purified and analyzed in respect to its biochemical and kinetic properties. In silico analysis of LipSm revealed that it is taxonomically related to several uncharacterized lipases from different genera, which constitute a unique clade, markedly different from all other previously described bacterial lipase families. All members of this clade displayed identical, conserved consensus sequence motifs, i.e. the catalytic triad (S, D, H), and an unusual, amongst bacterial lipases, Y-type oxyanion hole. 3D-modeling revealed the presence of a lid domain structure, which allows LipSm to act on small ester substrates without interfacial activation. In addition, the high percentage of alanine residues along with the occurrence of the AXXXA motif nine times in LipSm suggest that it is a thermostable lipase, a feature verified experimentally, since LipSm was still active after heating at 70 °C for 30 min.

**Conclusions:**

The phylogenetic analysis of LipSm suggests the establishment of a new bacterial lipase family (XVIII) with LipSm being its first characterized member. Furthermore, LipSm is alkaliphilic, thermostable and lacks the requirement for interfacial activation, when small substrates are used. These properties make LipSm a potential advantageous biocatalyst in industry and biotechnology.

**Electronic supplementary material:**

The online version of this article (10.1186/s40709-018-0074-6) contains supplementary material, which is available to authorized users.

## Background

Lipases (triacylglycerol acylhydrolase, E.C. 3.1.1.3) are enzymes, which hydrolyze triacylglycerols and release di- and/or monoglycerides, fatty acids and glycerol [1]. However, in low water content systems, they can also catalyze the reverse reactions i.e. esterifications or transesterifications [2, 3]. Due to these unique properties, lipases are enzymes of considerable biotechnological interest and find use in a broad spectrum of applications [4] including, among others, food technology, bioremediation, chemical industry and medical sciences [2, 5, 6].

Lipases are produced by both higher eukaryotes (plants and animals) and various microorganisms including fungi and bacteria [7]. Microbial lipases are reported to be advantageous when compared to enzymes of animal or plant origin, since they have a low cost–high production capacity, ease for genetic manipulation and are more stable in terms of activity [8]. These facts have led to an increased interest in the isolation of novel microbial lipases, especially for those, bearing biotechnological properties, which can be exploited in various industrial applications.

All lipases conform to *α*/*β* hydrolase fold [9] and at most share common structural and functional elements such as: (i) the catalytic triad (S, H and D), (ii) the conserved pentapeptide GXSXG in which the catalytic S is embedded, (iii) the mobile “lid” which shields the catalytic site, and (iv) the interfacial activation mechanism which regulates lid opening enabling the access of the substrates to the catalytic site [10, 11] and triggers the development of the so-called oxyanion hole [12]. However, despite their common features, lipases exhibit an extensive sequence variation. In the Lipase Engineering Database (LED) [12] lipases are assigned in three classes named GGGX, GX and Y according to the sequence and structure of the oxyanion hole [13].

However, when sequence identity and biochemical properties are considered, the diversity of these enzymes is much greater. Arpigny and Jaeger, classified first lipolytic enzymes in eight families (I–VIII) [14]. Since then, nine new families (IX–XVII) were further established [15–17]. Currently there are only two reports concerning the same *S. maltophilia* lipase gene (NCBI Accession No. KC014616), encoding LipSM54, a 60 kDa enzyme with particular features that established lipolytic family XVI [15, 18].

In this study, we present: (a) the cloning and overexpression of a novel *lipSm* gene from a new *S. maltophilia* strain, Psi-1, isolated from a sludge sample from a waste treatment facility at Psittaleia, Greece, (b) the biochemical and kinetic analysis of the purified lipase LipSm and (c) in silico analysis of the phylogenetic relationship of the encoded LipSm to other lipases, as well as its structural features, which provide evidence that LipSm is the first characterized member of a novel lipase family, indexed XVIII. To our knowledge, this is the first report on the production of an *S. maltophilia* lipase with such unusual structural and biochemical features.

## Results

### Selection and identification of the lipase producing strain Psi-1

**Fig. 1:**
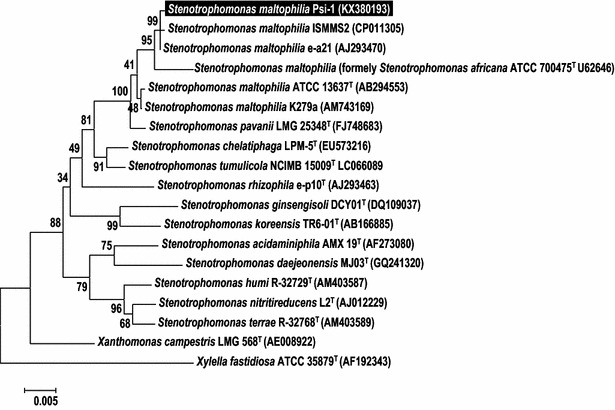
Phylogenetic position of *S*. *maltophilia* Psi-1 amongst other strains of *S*. *maltophilia* and related species. Phylogenetic analyses were conducted with algorithm MEGA. The tree was constructed with a neighbour joining algorithm using 16S rRNA gene sequences. *Xylella fastidiosa* served as an outgroup. The percentage of replicate trees in which the associated taxa clustered together in the bootstrap test (1000 replicates) are shown next to the branches. The scale bar indicates the number of substitutions per nucleotide position

Initial isolation on LA plates revealed the presence of 14 different morphological phenotypes in sludge samples collected from the waste treatment facilities of Volos and Psittaleia (Greece). Amongst them, only 5 phenotypes were found positive for lipase activity, using the true lipases’ specific Rhodamine B-olive oil agar plates assay, according to Kouker and Jaeger [19]. At least three isolates from each phenotype were preliminarily characterized using Intergenic Spacer (IGS) based molecular taxonomy. BLAST search revealed the presence of only one non-pathogenic species, belonging to *S. maltophilia* (data not shown). To verify the IGS-based taxonomic results, a strain designated Psi-1 (strain deposition number: LMG: 29922), randomly selected among the others, was further processed and its 16S rRNA gene was amplified, cloned in vector pBlueScript II KS (+) and sequenced. According to BLAST search results, Psi-1 was assigned to *S. maltophilia* species exhibiting a sequence homology of > 99% (GenBank Accession No. KX380193). The phylogenetic position of Psi-1 was traced in the phylogenetic tree derived from the 16S rRNA gene sequences of closely related taxa by a neighbor joining algorithm in MEGA software (Fig. 1).

### Amplification, cloning and sequencing of a putative secreted lipase gene (lipSm)

According to the bioinformatic analyses of the whole genome sequences of *S. maltophilia* strains deposited in the data banks, primers SMLF and SMLR were designed, to amplify a large DNA fragment (1850 bp) containing a putative secreted lipase gene, which was further cloned and sequenced. Based on these data, specific primers SMLPETF and SMLPETR were designed, in order to amplify and clone the putative *lipSm* ORF. Sequencing results verified the presence of the lipase gene (*lipSm*; 1203 bp; GenBank Accession No. KX353755) exhibiting its closest homology (91.8%) with the corresponding lipase gene from *S. maltophilia* strain K279a [20].

### Bioinformatic analysis of the deduced amino acid sequence of LipSm

**Fig. 2:**
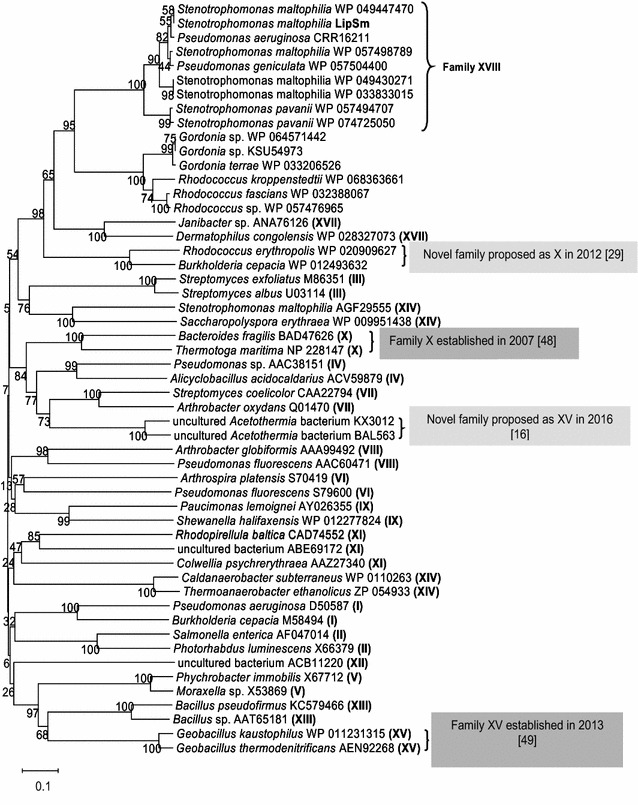
Phylogenetic dendrogram indicating the position of LipSm as the first characterized member of the novel family XVIII. Latin numbers in parentheses indicate family indexes. Phylogenetic analyses were conducted with algorithm MEGA. The evolutionary history was inferred using the Neighbor-Joining method. The percentage of replicate trees in which the associated taxa clustered together in the bootstrap test (1000 replicates) are shown next to the branches. Scale bar indicates substitutions per site

Analysis of the deduced amino acid sequence of LipSm (400 residues) revealed a predicted molecular mass (MM) of 42.08 kDa and a pI of 7.23. Pfam analysis classified LipSm as a secretory lipase, showing maximum identity at its C-terminus (amino acids 119–284). The presence of a signal peptide with a cleavage site located between residues 29 and 30 (LAA-AP) lends further support to the prediction of the extracellular location of the lipase, with the mature form (371 amino acid residues) exhibiting a calculated MM of 39.09 kDa and a pI value of 6.52. Sequence analysis of mature LipSm revealed the presence of the characteristic nucleophilic elbow (pentapeptide GH**S**QG), which includes the catalytic serine residue (S^154^). Furthermore, mature LipSm was found to contain a high percentage of alanine residues (16.7%) along with a 9-times presence of the motif AXXXA, which both have been reported as significant features of thermostable lipases [21]. BLAST searches revealed several non-redundant, putative, uncharacterized protein sequences showing high similarity to LipSm. Specifically, LipSm was found to share an up to 99% sequence identity with lipases from other strains of *S. maltophilia*. It also shares 93 and 91% identities with lipases from *Pseudomonas geniculata* and *P. aeruginosa*, respectively, as well as 86 and 87% identities with two lipases from *Stenotrophomonas pavanii*. Sequence identity of the novel LipSm with several actinobacterial lipases from *Gordonia* sp. and *Rhodococcus* sp. did not exceed 48%. For phylogenetic analysis, 38 lipolytic enzymes were selected as characteristic members of all existing bacterial lipase families along with 16 other proteins displaying identity with LipSm ranging from 45 to 99%. As depicted in Fig. 2, LipSm clusters together with the proteobacterial lipases from other *S. maltophilia* strains and the lipases from *P. geniculata* and *P. aeruginosa*. Under the same root are also found the *S. pavanii* lipases, however at a greater phylogenetic distance from LipSm, whereas the actinobacterial lipases constitute their own distinct, clearly distinguishable group. Figure 2 shows further that all other lipolytic families constitute different clades of the phylogenetic tree. Furthermore, BLAST search within LED [12] revealed a limited homology with lipases belonging to *Candida antarctica* lipase A like superfamily (abH38).

**Fig. 3:**
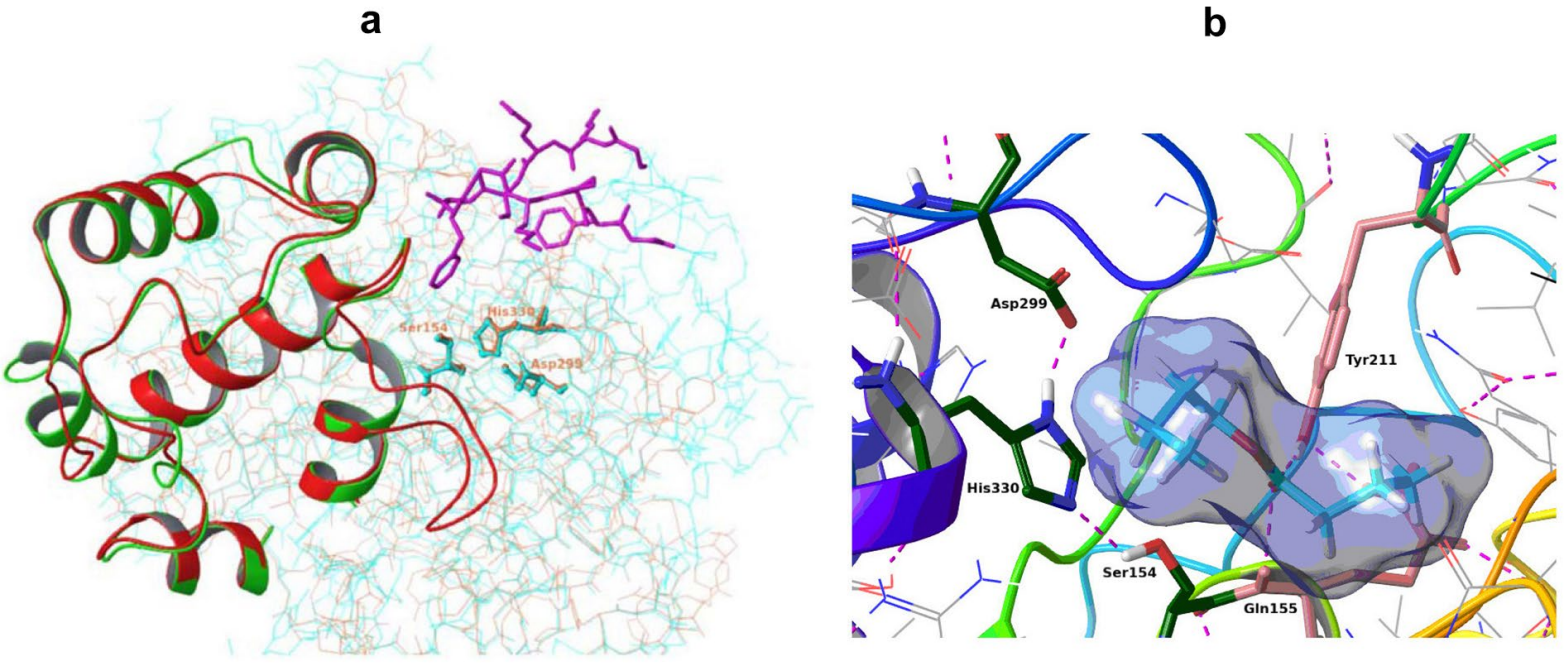
Structural depiction of LipSm. **a** Superimposition of 3D-structures of CALA (turquoise) and LipSm (brown) indicating their catalytic triads (S^154^, D^299^, H^330^; LipSm numbering) and highlighting both their lid domains, with green and red colors for CALA and LipSm, respectively, whereas in purple is shown the additional active-site flap of CALA (E^426^ up to E^436^) that LipSm lacks. **b** ES complex of LipSm with the substrate butyl-butyrate, where are illustrated the catalytic triad of LipSm, and its conserved residues Q^155^ and Y^211^, which form the Y-type oxyanion hole of LipSm. The hydrogen bonds are illustrated as purple dotted lines

**Fig. 4:**
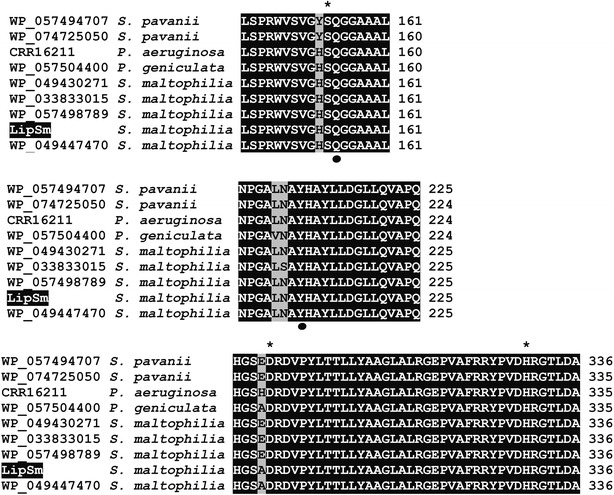
Blocks of conserved sequences amongst LipSm and other members of novel family XVIII. Identical and equivalent amino acids are on black or grey background, respectively. Stars and dots indicate amino acid residues constituting the catalytic triad and oxyanion hole, respectively

The 3D homology model constructed using the CALA (Protein Data Bank ID 2VEO) as a template, revealed a common α/*β* hydrolase fold. Despite the limited overall sequence identity of LipSm and CALA (ca. 23% as calculated by Clustal 2.1), the alignment (alignment score 0.050; RMSD 1.123 Å) of these two enzymes reveals a remarkable, structural similarity, which includes the catalytic triad (2VEO: S^184^, D^334^, H^366^; LipSm: S^154^, D^299^, H^330^) and the lid domain (2VEO: S^217^ up to E^308^; LipSm: A^188^ up to Y^279^), as depicted in Fig. 3a. Moreover, a consensus motif containing the conserved glutamine (Q^155^) and tyrosine (Y^211^) are located near the catalytic serine (S^154^), composing an unusual, among bacterial lipases, Y-type oxyanion hole, as it was shown during the docking studies (Fig. 3b). Apart from the extended sequence identity of LipSm with members of its clade (Fig. 2), a multiple alignment among them revealed a high degree of conservation of important structural/functional features such as the catalytic triad, and the oxyanion hole (Fig. 4; Additional file 1).

### Cloning, overexpression and purification of lipSm

**Fig. 5:**
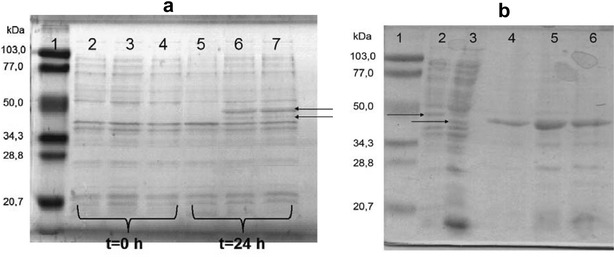
SDS-PAGE. **a** The whole cell protein extracts of two clones during overexpression, where in lane 1: MM marker; lanes 2, 5: BL21(DE3)/pET29c; lanes 3, 6: BL21(DE3)/pET29c::*lip* clone a; lanes 4, 7: BL21(DE3)/pET29c::*lip* clone b, **b** lipase purification steps, where in lane 1: MM marker; lane 2: crude protein extract at 24 h induction; lane 3: total soluble protein extract after cell lysis and centrifugation; lane 4: purified lipase LipSm fraction 1; lane 5: purified lipase LipSm fraction 2; lane 6: purified lipase LipSm fraction 3

Putative *lipSm* ORF was amplified accordingly to contain the restriction sites for *Nde*I (at its 5′-end) and *Xho*I, and subsequently cloned and overexpressed in an *E. coli* BL21(DE3)/pET29c(+) system. Interestingly, two bands appeared to be overexpressed on SDS polyacrylamide gels (Fig. 5a, indicated by arrows) corresponding to the MM of the premature enzyme form (44 kDa), as well as its mature form (40.7 kDa) generated after the removal of the signal peptide in *E. coli* BL21(DE3) cells (Fig. 5a). These molecular masses are in perfect agreement with the predicted ones of the premature LipSm enzyme and its mature form (42.08 and 39.09 kDa, respectively), when taken into account the additional 8 amino acid residues, originating from the inserted *Xho*I site and the C-terminus 6 × histidine-tag region. Mature LipSm was further purified from the total-soluble protein fraction of the recombinant *E. coli* BL21(DE3)/pET29c::*lipSm* clone as described in “Methods” section (Fig. 5b).

### Biochemical and kinetic analysis of LipSm

The novel lipase LipSm hydrolyzes the substrates Ac-4-NPh, Bu-4-NPh and La-4-NPh, exhibiting Michaelis–Menten kinetics in buffers lacking EDTAK_2_, while its activity was unaffected in the presence of DTT. However, the limited solubility of the two latter substrates in the used reaction buffer may have influenced the estimates of the corresponding Michaelis–Menten parameters (Table 1).

**Table 1:** Estimation of Michaelis–Menten parameters for the three substrates used in this study

Substrates	*k*_cat_/*K*_m_ (M^−1^ s^−1^)	*k*_cat_ (s^−1^)	*K*_m_ (mM)
4-Nitrophenyl acetate	3.671	1.736 × 10^−3^	0.473
4-Nitrophenyl butyrate	20.769	0.040	1.926
4-Nitrophenyl laurate	49.690	0.017	0.349

The Michaelis–Menten parameters of the novel lipase LipSm were evidently affected in the presence of higher concentrations of the salts CaCl_2_, MgCl_2_, MnCl_2_, and NaCl (hydrolysis of substrate Ac-4-NPh). In the case of [NaCl] = 10 mM, the values of both *k*_cat_/*K*_m_ and *k*_cat_ were decreased at about 50%, whereas the value of *K*_m_ remained unaffected; this is not uncommon. A negative effect was observed on the values of *k*_cat_/*K*_m_ and *k*_cat_ parameters, at both cases of high [CaCl_2_] and [MgCl_2_], whereas a positive effect was observed on the *K*_m_ values (~ 150%) for both salts; unambiguously, increased [CaCl_2_] and/or [MgCl_2_] in the enzymatic reaction mixture contributed in decreasing both the catalytic efficiency, and the turnover of LipSm, affecting also the lipase-substrate affinity. The effect of both low and high [MnCl_2_] in the reaction mixture was found almost similar; the observed decrease in the values of both *k*_cat_/*K*_m_ and *k*_cat_ was accompanied by no effect on the *K*_m_, more likely due to chelating effects which are easily formed by a multivalent transition element (Mn) (the effect of metallic salts on the Michaelis–Menten parameters of LipSm is depicted in more detail in Additional file 2).

**Fig. 6:**
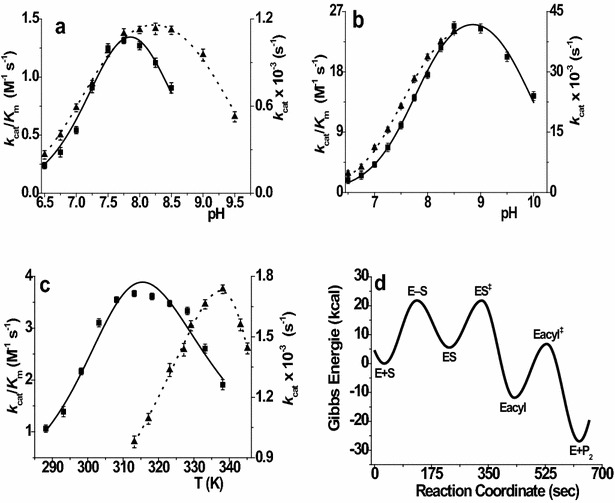
Kinetic analysis of LipSm. Merged curves of the dependencies of Michaelis–Menten parameters *k*_cat_/*K*_m_ (filled square solid lines) and *k*_cat_ (filled triangle dotted lines) versus the pH value, as well as versus the absolute temperature, and an energy diagram: **a** estimates of parameters *k*_cat_/*K*_m_ and *k*_cat_ versus the pH value, using 4-nitrophenyl acetate as substrate, where (*k*_cat_/*K*_m_)^lim^ = 2.33 ± 0.26 M^−1^ s^−1^, p*K*_a1_ = 7.42 ± 0.10 and p*K*_a2_ = 8.29 ± 0.10, as well as (*k*_cat_)^lim^ = 1.33 × 10^−3^ ± 0.03 × 10^−3^, p*K*_a1_ = 7.09 ± 0.03 and p*K*_a2_ = 9.36 ± 0.04; **b** estimates of parameters *k*_cat_/*K*_m_ and *k*_cat_ versus the pH value, using 4-nitrophenyl butyrate as substrate, where (*k*_cat_/*K*_m_)^lim^ = 29.24 ± 0.71 M^−1^ s^−1^, p*K*_a1_ = 7.78 ± 0.04 and p*K*_a2_ = 9.93 ± 0.05, as well as (*k*_cat_)^lim^ = 44.78 × 10^−3^ ± 0.87 × 10^−3^, p*K*_a1_ = 7.52 ± 0.02 and p*K*_a2_ = 10.33 ± 0.60; **c** the dependencies of parameters *k*_cat_/*K*_m_ and *k*_cat_ versus the absolute temperature, using 4-nitrophenyl acetate as substrate; **d** energy diagram of hydrolysis of substrate 4-nitrophenyl acetate by the novel lipase (the value of Arrhenius pre-exponential factor was assumed as 6 × 10^12^ s^−1^)

The profiles of parameters *k*_cat_/*K*_m_ and *k*_cat_ versus the pH value were obtained during the hydrolysis of substrates Ac-4-NPh and Bu-4-NPh by LipSm, and are depicted in Fig. 6a, b, respectively; in the case of substrate Ac-4-NPh (Fig. 6a) it is obvious that the enzyme species which correspond to the parameter *k*_cat_/*K*_m_ (i.e. the ES complex—narrower profile) is much less stable as compared to that corresponding to *k*_cat_ (i.e. the E_acyl_—broader profile). Moreover, it should be emphasized that the pH optimum value for the *k*_cat_ parameter is shifted to more alkaline values at about 0.38 pH units, versus that for the *k*_cat_/*K*_m_ parameter. These results are also supported from the estimated values of two p*K*_a_, in both cases of the *k*_cat_/*K*_m_ and *k*_cat_ parameters. In the case of *k*_cat_/*K*_m_, a p*K*_a_ value of 7.42 could be assigned to the formation of the ES complex (ionization of catalytic histidine), whereas a p*K*_a_ value of 8.29 could be assigned to the formation of an activated ES complex (tetrahedral intermediate) more likely due to the nucleophilic attack of the NH-group of the scissile amide bond onto the neutral imidazole of the catalytic histidine. Furthermore, in the case of *k*_cat_, a p*K*_a_ value of 7.09 could be assigned to the formation of the E_acyl_ species, whereas a p*K*_a_ value of 9.36 could be assigned to the transformation of E_acyl_ species to free enzyme and the product (acid). Furthermore, the profiles of parameters *k*_cat_/*K*_m_ and *k*_cat_ versus the pH value exhibit similar pH optima, in the case of the hydrolysis of substrate Bu-4-NPh, as it is depicted in Fig. 6b. These results imply that both enzyme species, i.e. the ES complex, as well as the E_acyl_, are equally stable, although there is a lack of experimental data in pH values > 8.5 due to the limited solubility of the used substrate in the reaction buffer.

In the latter case, the estimated values of the two p*K*_a_ (for both *k*_cat_/*K*_m_ and *k*_cat_ parameters) differ significantly from the corresponding p*K*_a_ values, which were estimated for the substrate Ac-4-NPh, and a similar assignment could be valid for these p*K*_a_ values, as in the case of substrate Ac-4-NPh, by taking into account also that the pH maxima in the hydrolysis of substrate Bu-4-NPh were shifted to more alkaline values (~ 8.87) [22, 23].

Moreover, the profiles of the parameters *k*_cat_/*K*_m_ and *k*_cat_ versus the absolute temperature at pH 8.00 were obtained and are depicted in Fig. 6c; LipSm exhibited optimum *k*_cat_/*K*_m_ and *k*_cat_ values at about 42.3 and 64.5 °C, respectively, while it maintained full activity up to 70 °C after 30 min of incubation, and then measuring its activity at 40 °C. These profiles were used to estimate the rate constants *k*_1_*, k*_−1_*, k*_2_ and *k*_3_, the corresponding activation energies, as well as the energy diagram (Fig. 6d). Furthermore, in agreement with the aforementioned results are the following i.e. (a) the profiles of *k*_cat_/*K*_m_ and *k*_cat_ versus the pH value showed that the ES complex is apparently higher at about 5.5 and 16.3 kcal from the species E + S and E_acyl_, respectively, and (b) the estimated values of ∆*H*^‡^, ∆*S*^‡^ and ∆*G*^‡^ for the parameters *k*_cat_/*K*_m_ and *k*_cat_, by mentioning out the effect on the entropies of activation during the formation of species ES and E_acyl_, respectively ($\Delta S_{{{{k_{\text{cat}} } /{K_{\text{m}} }}}}^{{^{\ddag } }} - \Delta S_{{k_{\text{cat}} }}^{\ddag }$ = 109.83 kJ mol^−1^ K^−1^).

In addition, the shift of the absolute temperature profile of *k*_cat_, towards higher temperatures, could verify that the reaction course from ES towards the products (substrate AC-4-NPh) dominates at higher temperatures; thus, the following relation are valid: *k*_cat_/*K*_m_ ≠ *k*_1_ and *k*_cat_/*K*_m_ = *k*_1_*k*_2_/(*k*_−1_ + *k*_2_) [24].

## Discussion

A new environmental *S*. *maltophilia* strain designated Psi-1 which exhibits lipase activity was isolated from a sample originating from a waste treatment facility. One of the strain’s lipase genes, designated *lipSm* and encoding a putative secreted lipase (LipSm) was successfully cloned and overexpressed in *E. coli*. LipSm, which was isolated, purified and analyzed biochemically and kinetically, is the second characterized lipase from *S. maltophilia* after the recently published *S. maltophilia* LipSM54 [15]. The two *S. maltophilia* lipase genes and their corresponding enzymes share low sequence identities, 62 and 23%, respectively. Furthermore, they exhibit distinct structural and biochemical properties, thus belonging to different lipase families (Fig. 2).

LipSm clustered together with the putative bacterial lipases from various strains of *S. maltophilia*, *P. geniculata* and *P. aeruginosa*, while *S. pavanii* lipases are also found in the same phylogenetic clade, albeit at a slightly greater phylogenetic distance (Fig. 2). Furthermore, important structural/functional features of these enzymes exhibit strong identity/similarity among themselves. These features, presented in Fig. 4, are: (i) the pentapeptide segment surrounding the catalytic serine residue (GH**S**QG for *S. maltophilia* strains, *P. geniculata* and *P. aeruginosa* and GY**S**QG for *S. pavanii* strains) and (ii) the conserved composition and positioning of the amino acid residues constituting the oxyanion hole.

Unfortunately, all other lipases belonging to the same clade as LipSm are uncharacterized and therefore no further comparison is possible. As depicted in Fig. 2, next to LipSm clade finds itself another clade consisting of putative actinobacterial lipases (*Gordonia* sp. and *Rhodococcus* sp.). This group of enzymes though, exhibits moderate sequence identities with LipSm (up to 48%). Furthermore, LipSm clade is clearly distal from any of the previously described lipase families (Fig. 2). These results suggest the classification of LipSm as the first characterized member of a novel bacterial lipase family, indexed XVIII.

Despite the low sequence similarity between the two enzymes, LipSm 3D homology model constructed using CALA (PDB ID 2VEO) as template, reveals a high semblance of the two structures, which includes the catalytic triad and the lid domain (Fig. 3a). However, it is noteworthy that LipSm lacks the additional active-site flap (2VEO: E^426^ up to E^436^), which is present in CALA [25]. The absence of the latter structure leads to a semi covered catalytic site for LipSm that allows the enzyme to act without interfacial activation, when small substrates are bound, as it was found in the docking studies. This was further experimentally substantiated by the fact that LipSm was active in reaction mixtures lacking surfactants (i.e. Triton X-100), when Ac-4-NPh or Bu-4-NPh were used as substrates (Fig. 6). Such microbial lipases that, despite having a lid, do not require interfacial activation with some substrates, are rare and include lipases from *P. aeruginosa* and *Burkholderia* (*Pseudomonas*) *glumae*, as well as, *C. antarctica* lipase B [26, 27]. It is noteworthy, that this novel enzyme has also an unusual Y-type oxyanion hole. Lipases, which contain the Y-type oxyanion hole, have been classified as Y-class lipases. To the Y-class have been assigned the *C. antarctica* lipase A [28] along with other fungal lipases of the superfamily (abH38). This structural feature of LipSm is unusual among bacterial lipases. The only other known bacterial lipases, which contain a Y-type oxyanion hole, as well as common structural features with CALA, are: (i) LipR and LipBX from *Rhodococcus* sp. and *Burkholderia cenocepacia*, respectively, both representatives of a distinct family proposed by Bassegoda et al. [29] and (ii) LipJ2 from *Janibacter* sp., the first characterized member of family XVII [17]. Despite these structural similarities, sequence identity of LipSm to the above mentioned characterized enzymes is low (20% to LipR and LipBX, approximately 25% to LipJ2), constituting clearly distinguishable families as depicted in Fig. 2.

In addition, emphasis should be given on the alkaliphilic and thermotolerant features, exhibited by this novel lipase during biochemical and kinetic studies. Regarding the pH effect on the activity of LipSm, it was proven that this enzyme possesses a slightly alkaliphilic character along with an augmented stability in its productive status, exhibiting its pH optima within the range of approximately 8–9 (Fig. 6a, b). These results are in perfect agreement with the optimum pH values reported for most lipases which lie between 7 and 9 [30]. In reference to LipSm thermostability, the enzyme maintained full activity up to 70 °C after 30 min of incubation. This experimental finding is further supported by in silico sequence analysis of LipSm that revealed the occurrence of the AXXXA motif nine times in the mature protein sequence, a structural signature of thermostable lipases since this motif is reported to cause better structural stability by strong van der Waals interaction in thermostable proteins [31, 32]. Furthermore, alanine residues in LipSm constitute 16.71% of the total amino acid composition, while the corresponding average value of a total of twenty-three microbial thermostable lipases, including *Pseudomonas* sp. lipases and CALA, is 8.88% [21]. The higher percentage of Ala is reported to attribute positively to lipase’s thermal stability [21].

## Conclusions

Lipases are enzymes which are used in a variety of biotechnological applications being able to catalyze a broad spectrum of reactions. This fact has led to an increased interest in the isolation of novel microbial lipases with particular properties, in order to be exploited as biocatalysts in various industrial applications. To this end, a novel lipase gene (*lipSm*) from *S. maltophilia* strain Psi-1 was cloned and overexpressed and the deduced enzyme LipSm was purified and characterized. The phylogenetic analysis of the novel enzyme suggests the establishment of a new bacterial lipase family (XVIII) with LipSm being its first characterized member. Furthermore, 3D modeling revealed that LipSm possesses a certain lid structure which enables it to act without the need for interfacial activation when small substrates are bound. This feature along with LipSm alkaliphilic and thermostable character, make LipSm a potential advantageous biocatalyst in industry and biotechnology.

## Methods

### Screening and isolation of lipase producing organisms

Sludge samples were collected from the waste treatment facilities in Volos and at Psittaleia (Greece) [33] and each cultivated in Luria–Bertani (LB) medium at 30 °C, for 24 h. Samples of the batch cultures were serially diluted on LA plates and incubated at 30 °C, for 24–48 h. Individual colonies with a distinct phenotype were removed and purified by repeated plate streaking, to obtain pure colonies and cultures. Representatives of each phenotype were further streaked on Rhodamine B-olive oil agar plates according to Kouker and Jaeger [19], in order to detect those, exhibiting lipase activity.

### Molecular techniques

Commercial kits Nucleospin Tissue, Nucleospin Plasmid and NucleoSpin Extract 2 in 1 were used to isolate genomic DNA, plasmid DNA and to extract DNA bands from agarose gels respectively, according to the suppliers’ recommendations (Macherey–Nagel, Düren, Germany). All Polymerase Chain Reactions (PCR) reactions were performed using the Kapa Hifi PCR System (KAPABIOSYSTEMS, Massachusetts, USA) according to the manufacturers’ recommendations, except for the variation of annealing temperatures whenever needed. All PCR primers were purchased from VBC-Biotech (Vienna, Austria). Vector pBlueScript II KS (+) (Stratagene, CA, USA) was used for all cloning experiments, while *Escherichia coli* (*E. coli*) strain DH5α served as the recombinant plasmid host [34].

Genomic DNA from *S. maltophilia* Psi-1 served as the template for PCR to amplify the 16S rDNA gene, using the primer pair 16SF (5′-AGTTTGATCCTGGCTCAG-3′) and 16SR (5′-AGAAAGGAGGTGATCCAGCC-3′) [35] and an annealing temperature of 60 °C. For the isolation of a large DNA fragment (1850 bp) containing a putative secreted lipase gene, primers SMLF (5′-AGTGGCCGAAGTACCCGTGG-3′) and SMLR (5′-CTGCCGGCCTATGACGTGCT-3′) were employed with an annealing temperature of 68 °C. The produced amplicon was cloned and the recombinant plasmid DNA served as a template to amplify the contained putative *lipSm* ORF. To this end, primer pair SMLPETF (5′-GAGCGCCATATGACCCCACCGCC-3′) and SMLPETR (5′-ATTCCTCGAGCGGGGACTCGTCCAGTACCTG-3′) containing restriction sites for *Nde*I and *Xho*I, respectively, was used to amplify *lipSm* ORF, at an annealing temperature of 70 °C. Cloned inserts were sequenced by CEMIA (Larissa, Greece). Taxonomic analysis was conducted using the GenBank BLAST program [36]. Phylogenetic and molecular evolutionary analysis was performed using MEGA software [37].

Finally, *lipSm* ORF was cloned in the pET29c(+) expression vector (Novagen, Wisconsin, USA) following restriction of both the expression vector and the gel-purified insert of clone pBlueScript II KS (+)/*lipSm* with *Nde*I–*Xho*I (Takara Bio Inc., Shiga, Japan). The resulting pET29c::*lipSm* construct was transformed into *E. coli* BL21(DE3) according to the method of Chung and Miller [38] for expression analysis.

### Amino acid sequence alignment, phylogeny, and structural analysis of LipSm

The web tool ORF Finder (http://www.ncbi.nlm.nih.gov/projects/gorf/) was used to identify the *lipSm* open reading frame (ORF). In silico translation to deduce the putative amino acid sequence (LipSm) corresponding to *lipSm* ORF was achieved by ExPASy translate tool [39]. Pfam web-tool [40] was used to assign LipSm to an enzyme class. SignaIP (version 4.1) software [41] was used to detect the occurrence of putative signal peptides. BLAST searches were performed to find the closest evolutionary relationships of LipSm [36]. ClustalOmega [42] was used to align LipSm with related sequences, whereas the phylogenetic position of LipSm was determined using the software MEGA [37]. A three-dimensional model for the target protein was generated using the SWISS-MODEL protein structure homology-modeling server [43]. Finally, computational analysis was applied in this work, on a Linux Platform, using the Schrödinger Software suite, ver. 2014-4 (Schrödinger LLC, Mannheim, Germany). The output generated by the SWISS-MODEL was further prepared by the Protein Preparation Wizard module. The molecular structure of the butyl-butyrate was built and prepared using the Build and the LigPrep module, respectively. Different conformations were generated with the Advanced Search of ConfGen module, by selecting water as solvent and the truncated Newton (TCNG) method for minimization (100 iterations). A 30 × 30 × 30 Å^3^ grid box with 10 × 10 × 10 Å^3^ default inner box was centered onto the catalytic triad (S^154^, D^299^, H^330^). All the output conformers were docked using the extra-precision (XP) mode of Glide docking module, and the top pose of the complex of LipSm with butyl-butyrate was used for the specification of oxyanion hole.

### Overexpression and purification of lipSm gene

Cells containing the pET29c::*lipSm* plasmid were incubated in 2 L LB containing kanamycin sulfate (Kn) (50 μg mL^−1^). The culture was incubated at 37 °C with aeration, in a 10 L Bioflo 110 bioreactor, equipped with a cooling/heating system (New Brunswick Scientific, Enfield, CT, USA). Culture was induced at an *A*_600_ of 0.6 by adding 0.5 mM isopropyl-β-d-1-thiogalactopyranoside (IPTG), and incubation was continued at 16 °C overnight. The cells were harvested by centrifugation, washed with 100 mM Tris–HCl, pH 8.0, and stored at − 20 °C [crude (whole cell) protein extract, lane 2 in Fig. 5b]. The cell pellet of the 2 L culture was thawed and resuspended in 20 mM sodium phosphate buffer, pH 7.6, containing 250 mM sodium chloride (NaCl) and 2% w/v TWEEN 20, at a ratio of approximately 5 mL buffer g^−1^ of dry cells. The suspension was subjected to ultrasonic treatment (10 pulses 10 s^−1^ 300 W^−1^ with interval pauses of 10 s for cooling the samples on ice) and centrifuged. The soluble supernatant fraction (total soluble protein extract, lane 3 in Fig. 5b) was loaded on a 5 mL HisTrap ff crude column using the ÄKTA FPLC protein purification system (GE Healthcare Bio-Sciences AB, Danderyd, Sweden), which was previously equilibrated with 5 column volumes (CVs) of an appropriate buffer (20 mM sodium phosphate buffer, pH 7.6, containing 250 mM NaCl, 1% w/v TWEEN 20 and 20 mM imidazole). In order to remove the unbound material, the column was washed with 10 CVs of the same buffer. Subsequently, LipSm was eluted in three successive steps using: (a) a 5 CVs length imidazole gradient (20–100 mM) of the aforementioned buffer, (b) 7 CVs length of the same buffer isocratic in imidazole (140 mM), and (c) 5 CVs length of the same buffer isocratic in imidazole but at higher concentration (500 mM) (Purified lipase LipSm Fractions, lanes 4–6 in Fig. 5b). Protein concentration was estimated by the method of Bradford [44] and protein profile was analyzed by Sodium Dodecyl Sulphate Polyacrylamide Gel Electrophoresis (SDS-PAGE) [45].

### Solutions, activity and kinetic measurements

All activity and kinetic measurements throughout this study were carried out in aqueous buffers of 100 mM Tris–HCl, pH 8.0 containing 1% Triton X-100 w/v at 40 °C, except, if otherwise stated. In all cases, the aqueous reaction media contained 5% v/v dimethylsulfoxide (DMSO), to increase the solubility of the used substrates [6, 23, 24, 46].

The kinetic measurements were performed spectrophotometrically by initial velocities at 405 nm, and at the appropriate pH and temperature values of the reaction mixtures, where the active concentration of LipSm was 850 nM for the substrates Ac-4-NPh and Bu-4-NPh, and 2200 nM for the substrate La-4-Nph. Each reaction run was initiated by adding 10 mL of the appropriate substrate solution in DMSO and the release of the leaving group (4-nitrophenol) was recorded; all kinetic measurements were repeated eight times.

### Effect of metallic salts, and dependencies of k_cat_/K_m_, k_cat_ and K_m_ of LipSm vs. pH and temperature

Kinetic measurements were performed at both low [S] (< 20 × *K*_m_) and at high [S] (> 5 × *K*_m_) of the substrate Ac-4-NPh in order to investigate the effect of metallic salts CaCl_2_, MgCl_2_, MnCl_2_, and NaCl on the Michaelis–Menten parameters of the novel lipase at various starting salt concentrations (1 and/or 10.0 mM), and estimates of the Michaelis–Menten parameters *k*_cat_/*K*_m_, *k*_cat_, and *K*_m_, were obtained. Additional measurements were performed using both substrates Ac-4-NPh and Bu-4-NPh at different pH values and at 40 °C, and then at varying temperatures and at pH 8.00, without 1% Triton X-100 w/v, as previously described [23]. The thermo tolerance of LipSm was tested by incubating the corresponding enzyme preparations at a range of temperatures (20 °C up to 70 °C), for 30 min, and then by performing activity measurements using these preparations at the temperature of 40 °C.

### Analysis of data

The experimental data of the dependencies of Michaelis–Menten parameters *k*_cat_/*K*_m_, *k*_cat_ and *K*_m_ versus the substrate concentration, pH values and absolute temperature, were analyzed by means of the familiar Michaelis–Menten equation, as well as by Eqs. (1)–(4) [46, 47]. In these equations, *k*_obs_ and (*k*)^lim^ are referred to either parameter *k*_cat_/*K*_m_ or *k*_cat_.

1 $$k_{\text{obs}} = \frac{{\left( k \right)^{\lim } }}{{1 + 10^{{{\text{p}}K_{{{\text{a}}1}} - {\text{pH}}}} + 10^{{{\text{pH}} - {\text{p}}K_{\text{a2}} }} }}$$

2a $${\text{for}}\;{{k_{\text{cat}} }/ {K_{\text{m}} }}{:}\;\frac{{k_{\text{cat}} }}{{K_{\text{m}} }} = \left\{ {\frac{{{{\upalpha}}_{0} {\text{e}}^{{\left[ {\frac{{{\text{E}}_{{\upalpha}} }}{\text{R}}\left( {\frac{1}{\text{T}} - \frac{1}{{{\text{T}}_{0} }}} \right)} \right]}} }}{{1 + {{\upalpha}}_{0} {\text{e}}^{{\left[ {\frac{{{\text{E}}_{{\upalpha}} }}{\text{R}}\left( {\frac{1}{\text{T}} - \frac{1}{{{\text{T}}_{0} }}} \right)} \right]}} }}} \right\}\left( {k_{1} } \right)_{0} {\text{e}}^{{\left[ { - \frac{{{\text{E}}_{1} }}{\text{R}}\left( {\frac{1}{\text{T}} - \frac{1}{{{\text{T}}_{0} }}} \right)} \right]}}$$

2b $${\text{and}}\;\frac{{k_{\text{cat}} }}{{K_{\text{m}} }} = \frac{{\left( {k_{1} } \right)_{0} \left( {k_{2} } \right)_{0} {\text{e}}^{{\left[ {\frac{{ - \left( {{\text{E}}_{1} + {\text{E}}_{1} } \right)}}{\text{R}}\left( {\frac{1}{\text{T}} - \frac{1}{{{\text{T}}_{0} }}} \right)} \right]}} }}{{\left( {k_{ - 1} } \right)_{0} {\text{e}}^{{\left[ { - \frac{{{\text{E}}_{ - 1} }}{\text{R}}\left( {\frac{1}{\text{T}} - \frac{1}{{{\text{T}}_{0} }}} \right)} \right]}} + \left( {k_{2} } \right)_{0} {\text{e}}^{{\left[ { - \frac{{{\text{E}}_{2} }}{\text{R}}\left( {\frac{1}{\text{T}} - \frac{1}{{{\text{T}}_{0} }}} \right)} \right]}} }}$$

3 $${\text{for}}\;k_{\text{cat}}{:}\;k_{\text{cat}} = \frac{{\left( {k_{2} } \right)_{0} \left( {k_{3} } \right)_{0} {\text{e}}^{{\left[ {\frac{{ - \left( {{\text{E}}_{2} + {\text{E}}_{3} } \right)}}{\text{R}}\left( {\frac{1}{\text{T}} - \frac{1}{{{\text{T}}_{0} }}} \right)} \right]}} }}{{\left( {k_{2} } \right)_{0} {\text{e}}^{{\left[ { - \frac{{{\text{E}}_{2} }}{\text{R}}\left( {\frac{1}{\text{T}} - \frac{1}{{{\text{T}}_{0} }}} \right)} \right]}} + \left( {k_{3} } \right)_{0} {\text{e}}^{{\left[ { - \frac{{{\text{E}}_{3} }}{\text{R}}\left( {\frac{1}{\text{T}} - \frac{1}{{{\text{T}}_{0} }}} \right)} \right]}} }}$$

4 $${\text{Eyring}}\;{\text{equation: T}} \times \left[ {\ln \left( {{k/{\text{T}}}} \right)} \right] = {\text{T}} \times \left[ {\ln \left( {{{k_{B} } / \hbar }} \right) + {{\Delta S^{\ddag } } / R}} \right] - {{\Delta H^{\ddag } } / R}$$

## Additional files

**Additional file 1.** Complete sequence alignments among mature forms of LipSm and members of its clade. Protein sequences exhibit identities with each other ranging between 85 and 98% (as calculated by Clustal2.1).

**Additional file 2.** Effect of metallic salts on the Michaelis–Menten parameters of LipSm.

## Abbreviations

CALA: *Candida antarctica* lipase A
Ac-4-NPh: 4-nitrophenyl acetate
Bu-4-NPh: 4-nitrophenyl butyrate
La-4-NPh: 4-nitrophenyl laurate
OFR: open reading frame
