# Correction to: Molecular, biochemical and kinetic analysis of a novel, thermostable lipase (LipSm) from *Stenotrophomonas maltophilia* Psi‑1, the first member of a new bacterial lipase family (XIX)

**DOI:** 10.1186/s40709-018-0083-5

**Published:** 2018-06-14

**Authors:** Maria Parapouli, Athanasios Foukis, Panagiota‑Yiolanda Stergiou, Maria Koukouritaki, Panagiotis Magklaras, Olga A. Gkini, Emmanuel M. Papamichael, Amalia‑Sofia Afendra, Efstathios Hatziloukas

**Affiliations:** 1 Enzyme Biotechnology and Genetic Engineering Group, University of Ioannina, 451 10 Ioannina, Greece; 2 Department of Biological Applications & Technologies, University of Ioannina, University Campus, 451 10 Ioannina, Greece

## Correction to: J Biol Res-Thessaloniki (2018) 25:4 10.1186/s40709-018-0074-6

We have recently (8th February 2018) published our article entitled “Molecular, biochemical and kinetic analysis of a novel, thermostable lipase (LipSm) from *Stenotrophomonas maltophilia* Psi-1, the first member of a new bacterial lipase family (XVIII)” [1]. While our manuscript was going through the final stages of publication, an article by Samoylova et al. [2] was published (12th January 2018) in the journal *Extremophiles*, entitled “Cloning, expression and characterization of the esterase estUT1 from *Ureibacillus thermosphaericus* which belongs to a new lipase family XVIII”. Since we could not have known of the work of Samoylova et al. [2] when we submitted our manuscript, and in order to avoid confusion in the scientific community, we propose to reclassify LipSm as the first characterized member of the new bacterial lipase family XIX. Therefore throughout our article [1] “lipase family XVIII” should read “lipase family XIX” (title included).
