# Supplementary material for: Molecular, biochemical and kinetic analysis of a novel, thermostable lipase (LipSm) from Stenotrophomonas maltophilia Psi-1, the first member of a new bacterial lipase family (XVIII)
Source: J Biol Res (Thessalon). 2018 Feb 8;25:4. doi: 10.1186/s40709-018-0074-6 (PMC5806266; doi:10.1186/s40709-018-0074-6)
Supplement: Supplementary file 1 — Additional file 1. Complete sequence alignments among mature forms of LipSm and members of its clade. Protein sequences exhibit identities with each other ranging between 85 and 98% (as calculated by Clustal2.1). [file 40709_2018_74_MOESM1_ESM.doc]

WP_057494707 ARDAPGDLLSAAPYRAPWVPSRAAQAYKLHYRTPDHHGALAEGTGLLYLPAGTPPIGGWP 60

WP_074725050 -RGAPGDLLSAAPYRAPWVPSRAAQAFKLHYRTPDHHGALAEGTGLLYLPAGTPPIGGWP 59

CRR16211 -QGAPGDLLSAAPYRATWVPSQAAQAYKLHYRTPDHRGQLAEGTGLLYLPAGTPPIGGWP 59

WP_057504400 APVAPGDLLSAAPYRASWVPSKAAQAYKLHYRTPDHRGQLAEGTGLLYLPAGAAPAGGWP 60

WP_049430271 ASPAPGDLLSAAPYRASWVPSKAAQAYKLRYRTPDHRGQLAEGAGLPYLPAGAAPAGGWP 60

WP_033833015 ASPAPGDLLSAAPYRASWVPSKAAQAYKLRYRTPDHRGQLAEGAGLPYLPAGAAPAGGWP 60

WP_057498789 APAAPGDLLSAAPYRASWVPSKAAQAYKLQYRTPDHRGQLAEGTGLLYLPAGAVPAGGWP 60

LipSm APVAPGDLLSAAPYRASWVPSKAAQAYKLHYRTPDHRGQLAEGTGLLYLPAGAAPAGGWP 60

WP_049447470 APVAPGDLLSAAPYRASWVPSKAAQAYKLHYRTPDHRGQLAEGTGLLYLPAGAAPAGGWP 60

************* ****:****:**:******:* ****:** *****: * ****

WP_057494707 VVSWAHGTQGIADRCAPSVSGPTHAERDGRFLDVFLAQGYAVAAADYQGLGSPGRHAYLH 120

WP_074725050 VVSWAHGTQGIADRCAPSVSGPTHAERDGRFLDVFLAHGYAVAAADYQGLGSPGRHAYLH 119

CRR16211 VVSWAHGTQGIADRCAPSVSGPYQPERDGRFLDRFLAQGYAVVAADYQGLGSSGDHAYLH 119

WP_057504400 VVSWAHGTQGIADRCAPSVSGPYQPQRDGRFLDQFLAQGYAVVAADYQGLGSPGDHAYLH 120

WP_049430271 VVSWAHGTQGIADYCAPSVSGPYQPERDGRFLDQFLAQGYAVVAADYQGLGSPGDHAYLH 120

WP_033833015 VVSWAHGTQGIADYCAPSVSGPYQPERDGRFLDQFLAQGYAVVAADYQGLGSPGDHAYLH 120

WP_057498789 VVSWAHGTQGIADRCAPSVSGPYQPERDGRFLDQFLAQGYAVVAADYQGLGSPGDHAYLH 120

LipSm VVSWAHGTQGIADRCAPSVSGPYQPERDGRFLDQFLAQGYAVVAADYQGLGNPGDHAYLH 120

WP_049447470 VVSWAHGTQGIADRCAPSVSGPYQPERDGRFLDQFLAQGYAVVAADYQGLGNPGDHAYLH 120

************* ******** : :******* ***:****.********. * *****

WP_057494707 VRTAARNTIDMVKASRQYLGTAALSPRWVSVGYSQGGAAALVAGHLAASHGGPSLQYRGS 180

WP_074725050 VRTAARNTIDMVKASRQYLGAAALSPRWVSVGYSQGGAAALVAGHLAASHGGPSLQYRGS 179

CRR16211 VRTAARNAIDMIKASRQYLGNGTLSPRWVSVGHSQGGAAALTAGHIAPTYGGPSLQYRGS 179

WP_057504400 VRTAARNAIDLVKASRQYLGNA-LSPRWVSVGHSQGGAAALTAGHIAPTHGGPALHYRGS 179

WP_049430271 VRAAARNAIDLVKASRQYLGTATLSPRWVSVGHSQGGAAALTAGHLAPTYAGPSLHYRGS 180

WP_033833015 VRAAARNAIDLVKASRQYLGTATLSPRWVSVGHSQGGAAALTAGHLAPTYAGPSLHYRGS 180

WP_057498789 VRTAARNAIDLVKASRQYLGNATLSPRWVSVGHSQGGAAALTAGHIAPTYGGPSLHYRGS 180

LipSm VRTAARNAIDLIKASRQYLGTATLSPRWVSVGHSQGGAAALTAGHIAPTYGGPALHYRGS 180

WP_049447470 VRTAARNAIDLIKASRQYLGTATLSPRWVSVGHSQGGAAALTAGHIAPTYGGPALHYRGS 180

**:****:**::******** . *********:********.***:* ::.**:*:****

WP_057494707 FTAGTPTAVELTALVMKPDNRTANPGALNAYHAYLLDGLLQVAPQVDRVLSDAGRARIAL 240

WP_074725050 FTAGTPTAVELTALVMKPDNRTANPGALNAYHAYLLDGLLQVAPQVDRVLSDAGRARIAL 239

CRR16211 FTTGTPTAVELTALVMKPDNRTANPGALNAYHAYLLDGLLQVAPQIDRVLSDAGRARVAV 239

WP_057504400 FTTGTPTAVELTALVMKPDNRSPNPGAVNAYHAYLLDGLLQVAPQIDRVLSDTGRARVAA 239

WP_049430271 FTTGTPTAVDLTALVMKPDNRIANPGALNAYHAYLLDGLLQVAPQIDRVLSDTGRARVAV 240

WP_033833015 FTTGTPTAVDLTALVMKPDNRIANPGALSAYHAYLLDGLLQVAPQIDRVLSDTGRARVAV 240

WP_057498789 FTTGTPTAVELTALVMKPDNRTANPGALNAYHAYLLDGLLQVAPQIDRVLSDTGRARVAV 240

LipSm FTTGTPTAVELTALVMKPDNRTANPGALNAYHAYLLDGLLQVAPQIDRVLSDTGRARVAV 240

WP_049447470 FTTGTPTAVELTALVMNPDNRTANPGALNAYHAYLLDGLLQVAPQIDRVLSDTGRARVAV 240

**:******:******:**** ****:.****************:******:****:*

WP_057494707 AREQCLGDLASTLDGMDTGSMFTAPLTGVPGIWTVLHDYLGVPRRGFSQPLMLAHGSEDR 300

WP_074725050 AREQCLGDLASTLDGMDTGSMFTAPLTGVPGIWTVLHDYLGVPRRGFSQPLMLAHGSEDR 299

CRR16211 AREQCLGELATTLDGADTGSMFTAPLASVPGIWAVLYDYLGVPRRGFSQPLMLGHGSHDR 299

WP_057504400 AREQCLGELAATLDGADIGSMFTAPLTSVPGIWAVLYDYLGVPRRGFSQPLMLGHGSADR 299

WP_049430271 AREQCLGELATTLDGADTGSMFTAPLTSVPGIWAVLYDYLGVPRRGFSQPLILGHGSEDR 300

WP_033833015 AREQCLGELATTLDGADTGSMFTAPLTSVPGIWAVLYDYLGVPRRGFSQPLILGHGSEDR 300

WP_057498789 AREQCLGELATTLEGADTGSMFTEPLTSVPGIWAVLYDYLGVPRRGFSQPLMLGHGSEDR 300

LipSm AREQCLGELAATLDGADTGSMFTAPLTSVPGIWAVLYDYLGVPRRGFSQPLMLGHGSADR 300

WP_049447470 AREQCLGELAATLDGADTGSMFTAPLSSVPGIWAVLYDYLGVPRRGFSQPLMLGHGSADR 300

*******:**:**:* * ***** **:.*****:**:**************:*.*** **

WP_057494707 DVPYLTTLLYAAGLALRGEPVAFRRYPVDHRGTLDAAAADGLAFVRARLEDDAFNDAAEI 360

WP_074725050 DVPYLTTLLYAAGLALRGEPVAFRRYPVDHRGTLDAAAADGLAFVHARLEDDPFNDTAEI 359

CRR16211 DVPYLTTLLYAAGLALRGEPVAFRRYPVDHRGTLDAAAADGLGFVRARLGDDPFNDAAET 359

WP_057504400 DVPYLTTLLYAAGLALRGEPVAFRRYPVDHRGTLDAAAADGLAFVRARLEGGHFSDTAET 359

WP_049430271 DVPYLTTLLYAAGLALRGEPVAFRRYPVDHRGTLDA-AADGLAFVRARLEDGHFNDAAET 359

WP_033833015 DVPYLTTLLYAAGLALRGEPVAFRRYPVDHRGTLDA-AADGLAFMRARLEDGHFNDAAET 359

WP_057498789 DVPYLTTLLYAAGLALRGEPVAFRRYPVDHRGTLDAAAADGLAFVRARLGDGHFNDAAET 360

LipSm DVPYLTTLLYAAGLALRGEPVAFRRYPVDHRGTLDAAAADGLAFVRARLGDAHFNEAAET 360

WP_049447470 DVPYLTTLLYAAGLALRGEPVAFRRYPVDHRGTLDAAAADGLAFVRARLGDAHFNEAAET 360

************************************ *****.*::*** . *.::**

WP_057494707 AGLEQVLDEAR 371

WP_074725050 AGLEQMLDEAR 370

CRR16211 AGLEQLLDEVG 370

WP_057504400 AGLEQLLDEAR 370

WP_049430271 AGLEQLLDDAR 370

WP_033833015 AGLEQLLDDAR 370

WP_057498789 AGLEQLLDDAR 371

LipSm AHLEQVLDESP 371

WP_049447470 AHLEQVLDESP 371

* ***:**:

Additional file 1
